# Decrease of visits and hospital admissions for cancer patients during the COVID-19 pandemic. A systematic review and meta-analysis

**DOI:** 10.1007/s10389-023-01857-w

**Published:** 2023-03-16

**Authors:** Marco Angelini, Federica Teglia, Giulia Casolari, Laura Astolfi, Paolo Boffetta

**Affiliations:** 1 Department of Medical and Surgical Sciences, University of Bologna, Via Massarenti 9, 40138 Bologna, Italy; 2 Stony Brook Cancer Center, Stony Brook University, New York, NY USA

**Keywords:** COVID-19 pandemic, Hospital admissions, Meta-analysis

## Abstract

**Background:**

During the COVID-19 pandemic, many nonurgent oncologic services were postponed. The aim of the present study was to estimate the impact of the pandemic on visits and hospital admissions for cancer patients worldwide.

**Methods:**

In our systematic review and meta-analysis, databases such as Pubmed, Proquest, and Scopus were searched comprehensively for articles published between January 1, 2020, and December 12, 2021. We included articles reporting data comparing the number of visits and hospital admissions for oncologic patients performed before and during the pandemic. Two pairs of independent reviewers extracted data from the selected studies. The weighted average of the percentage change was calculated and compared between pandemic and pre-pandemic periods. Stratified analysis was performed by geographic area, time interval, and study setting.

**Findings:**

We found a mean relative change throughout January–October 2020 of –37.8% (95% CI –42.6; –32.9) and –26.3% (95% CI –31.4; –21.1) compared to pre-pandemic periods for oncologic visits and hospital admission, respectively. The temporal trend showed a U-shaped curve with nadir in April for cancer visits and in May 2020 for hospital admissions. All geographic areas showed a similar pattern and the same was observed when stratifying the studies as clinic-based and population-based.

**Interpretation:**

Our results showed a decrease in the number of visits and hospital admission during the January–October 2020 period after the outbreak of the COVID-19 pandemic. The postponement or cancellation of these oncologic services may negatively affect the patient’s outcome and the future burden of disease.

**Supplementary Information:**

The online version contains supplementary material available at 10.1007/s10389-023-01857-w.

## Introduction

The Coronavirus disease 2019 (COVID-19) pandemic caused an extraordinary burden on the healthcare system and dramatically impacted the delivery of medical services. During the acute phase of the pandemic, many nonurgent oncologic services were deferred (Teglia et al. 2022a, Patt et al. 2020), and the number of patients accessing hospitals for emergency reasons was lower compared to the previous year, according to the National Health Service of England (2021). The causes for this decline must still be determined, but they are likely to be multifactorial.

The pandemic brought up the need for a systemic reorganization of health systems in order to protect both medical personnel and incoming patients from the risk of in-hospital infection and to provide the required care to COVID-19 patients, especially the ones at greater risk, such as the oncologic ones (Di Felice et al. 2022). Uncertainty was mainly derived from concerns about cancer progression and a negative impact on survival, which contributed to a sense of urgency to provide the right treatment at the right moment (Turaga and Girotra 2020). Notwithstanding, non-emergency clinical services were deprioritized (Sud et al. 2020), leading to significant concern among specialists caring for patients with both early and advanced cancer.

Avoiding care of diseases requiring timely treatment may have had significant clinical and public health consequences. It has been shown that during the pandemic hospitalizations for emergency and potentially life-threatening conditions significantly declined, possibly due to a combination of factors, such as patients ignoring symptoms, obeying stay-at-home orders, and fear of getting infected at hospitals (Lee et al. 2020, Repici et al. 2020).

We performed a systematic review and meta-analysis of studies that analyzed the relative change in the total number of medical visits and hospital admissions for cancer patients from the beginning of the pandemic in March 2020 compared to the pre-pandemic period.

## Materials and methods

### Search strategy and selection criteria

**Fig. 1:**
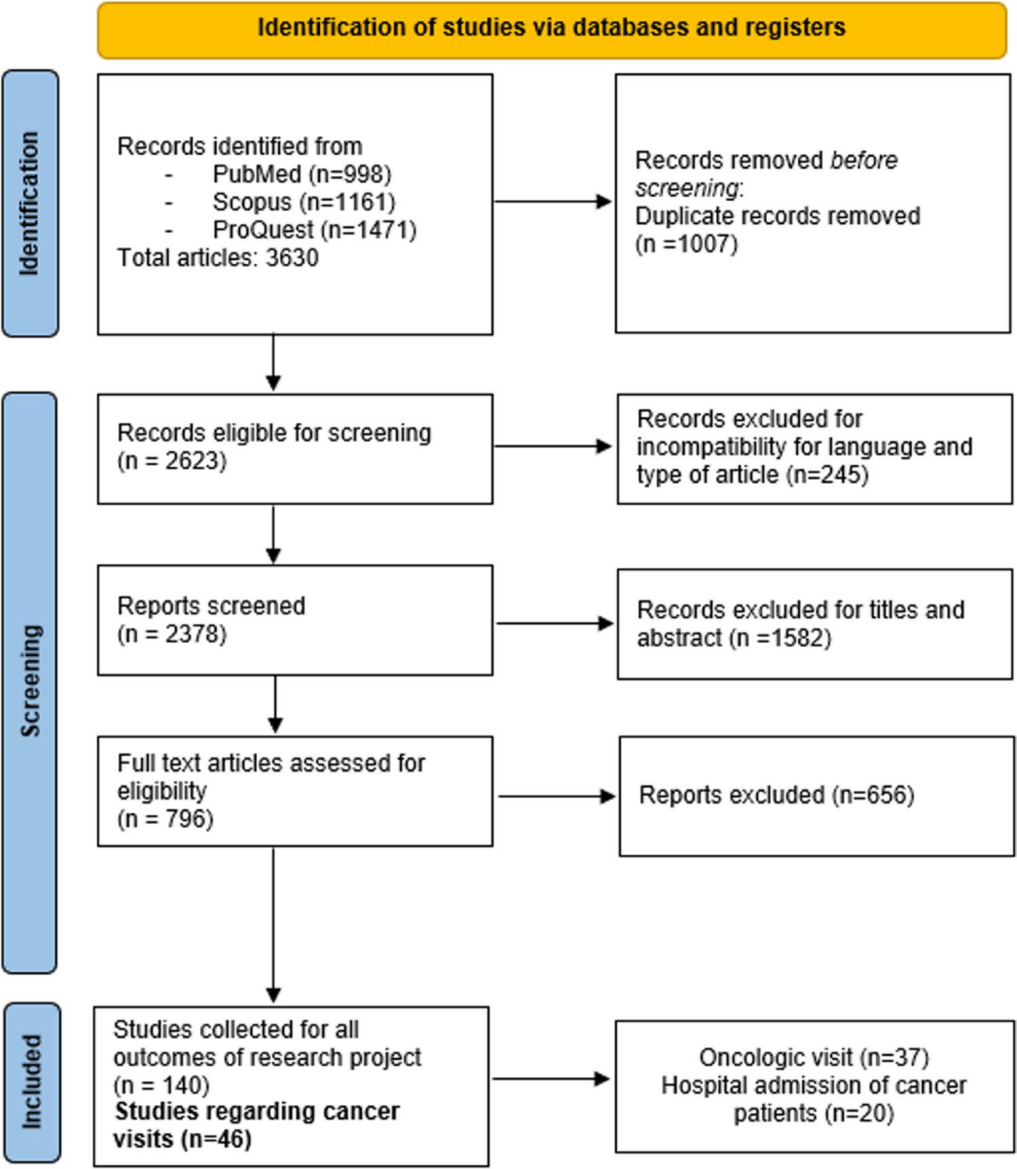
PRISMA (Preferred Reporting Items for Systematic Reviews and Meta-analyses) flow diagram

The research protocol of the systematic review and meta-analysis, conducted according to the PRISMA statement (Fig. 1) (Moher et al. 2009), was included in the PROSPERO Register (registration number CRD42022314314). The study was developed following the patients or problem, intervention, comparison group, outcomes and study design (PICO) framework. Since this study is part of a larger project aiming at assessing the global impact of the COVID-19 pandemic on cancer care, including cancer screening (Teglia et al. 2022a), oncologic treatment (Teglia et al. 2022b), diagnostic tests, and diagnosis of cancer (Angelini et al. 2023). For this reason, the search strategy and the selection criteria were extensively explained in the first paper published (Teglia et al. 2022a) on the matter, to which reference can be made for further details. Briefly, we included in our study articles reporting quantitative relative changes in the number of visits and hospital admission for cancer patients, performed before and after the beginning of the COVID-19 pandemic.

### Data collection and quality assessment

The process of article identification, selection and inclusion in our study has been detailed elsewhere (Teglia et al. 2022a). For the analysis of the present outcome, we retained 46 articles: 37 for oncologic visits and 20 for hospital admission of cancer patients (including 11 articles reporting results for both). We finally performed a quality assessment of all the studies included in our review using the Critical Appraisal Skills Programme (CASP 2018) score for qualitative research. Studies obtaining less than 7 points were considered inadequate and excluded from the meta-analysis (no article was excluded due to low quality score). Supplementary Table 1 and 2 list the studies included in the present analysis, their major characteristics and quality assessment.

### Statistical analyses

The methods of the statistical analysis have been thoroughly explained in the first article of the research project (Teglia et al. 2022a). Briefly, we calculated the weighted average relative change of the number of daily events (medical visits and hospital admissions, separately) between the two periods (one before and one after the beginning of the COVID-19 pandemic). In doing so, we divided the pandemic period into five time intervals (January–February 2020, March 2020, April 2020, May 2020, and June–October 2020). We performed additional stratification by geographic area and by type of setting of the study. These variables were fitted into an ordinary least-squared linear model analysis using Newton–Raphson (maximum likelihood) optimization with 2-sided *P* values, considering *P* < .05 statistically significant.

Many studies reported data about different periods. In order to avoid counting the same article multiple times, we used the mean value if the variables were repeated within the same article. For example, if an article reported data for three different time intervals, we used their weighted mean to assess the relative change when all periods were considered together.

We performed the Q-statistic, which compares the variability between the effect sizes of studies with the amount of variation expected when the studies estimate the same effect; then we analyzed heterogeneity across studies using the I^2^ test. Then we used the funnel plot and the Egger’s regression asymmetry test to assess publication bias (Egger et al. 1997). No ethics committee approval was necessary because the study was restricted to publicly available data. For all statistical analyses, we used STATA version 16.1 (Stata Corp., College Station, TX, US). This research was supported by internal resources of the participating institutions.

## Results

### Visits

**Fig. 2:**
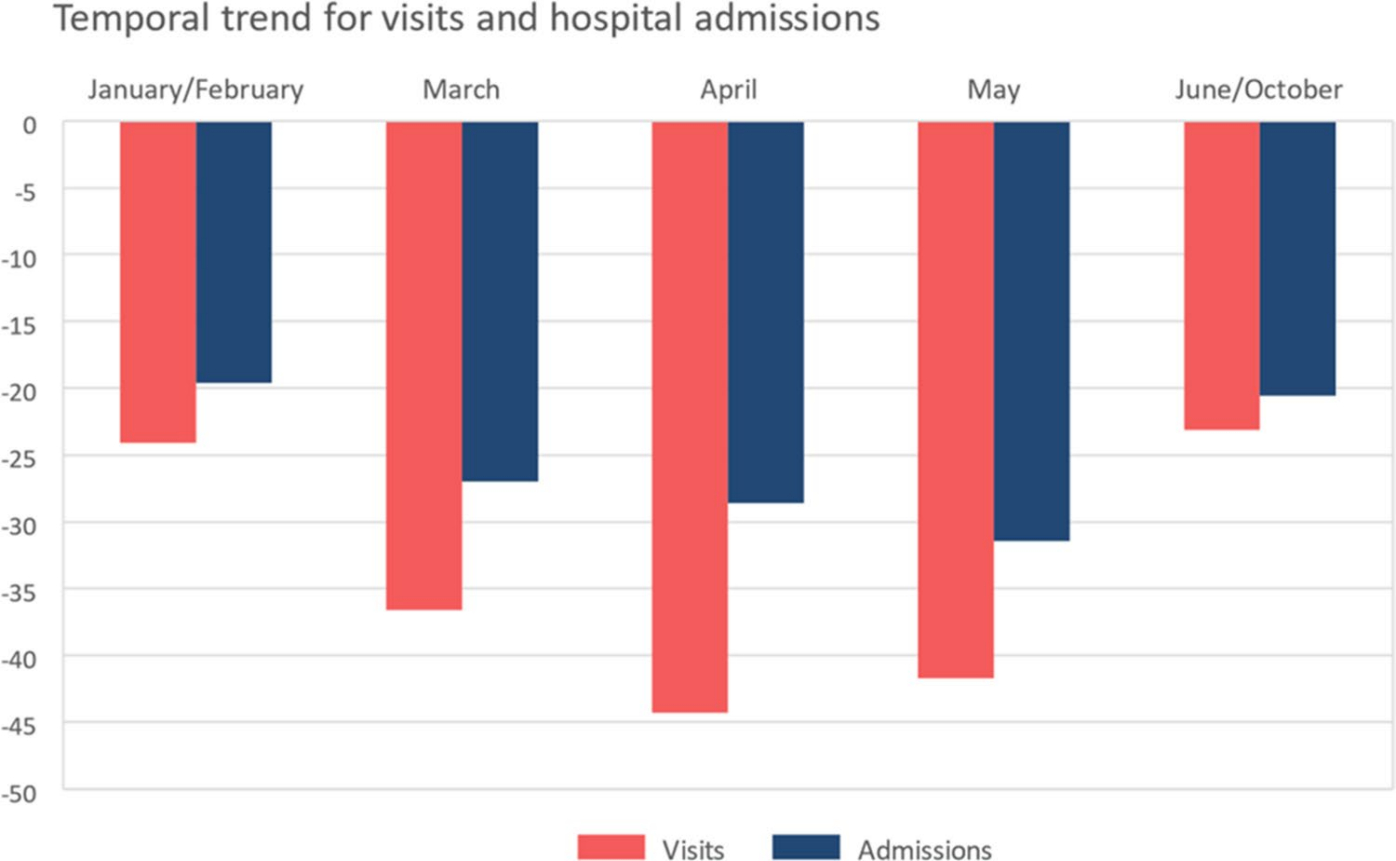
Temporal trend of the weighted average percentage variation of visits and hospital admission for cancer patients from January to October 2020 compared to the pre-pandemic period, divided by time-interval

The mean change for oncologic visits throughout January–October 2020 compared to the pre-pandemic period was –37.8% (95% CI –42.6; –32.9), which reached its maximum decline in April (–44.3%, 95% CI –55.9; –32.8) and showed a significant reduction up to the last period of analysis (June–October: –23.1%, 95% CI –35.0; –11.3) (Fig. 2). When considering only first oncologic visits, the relative change was –33.5% (95% CI –41.6; –29.6); follow-up visits showed a larger decline, equal to –45.4% (95% CI –57.6; –33.3) (Table 1). All geographic areas showed a similar decline: –40.4%, –39.0%, –33.4% and –37.8% for North America, Europe, Latin America, and Asia, respectively (Table 1; the distribution of the data by geographic area is shown in Supplementary Fig. 1). Clinic-based and population-based studies showed a comparable reduction: –39.5% (95% CI –46.6; –32.3) and –36.9% (95% CI –44.1; –29.6), respectively (Table 1). The multivariate linear analysis displayed in Table 2 confirmed these results, showing no significant differences by types of visit, period, geographic area, and study setting.

**Table 1:** Weighted percentage variation of overall, first and follow-up visits and of hospital admissions for cancer patients from January to October 2020 compared to pre-pandemic period, divided by period, geographic area, and study setting

Characteristic	Overall visits	First visits	Follow-up visits	Admissions
Period (2020)				
January and February	–24.1 (–40.4; –7.7)	–21.1 (–76.2; 34.1)	/	–19.6 (–62.6; 23.5)
Mar	–36.6 (–42.9; –30.3)	–35.3 (–45.6; –24.9)	–37.6 (–54.0; –21.1)	–27.0 (–32.4; –21.5)
Apr	–44.3 (–55.9; –32.8)	–33.8 (–48.6; –19.0)	–48.0 (–81.6; –14.4)	–28.6 (–66.9; 9.7)
May	–41.7 (–54.2; –29.3)	–37.8 (–60.1; –15.5)	/	–31.5 (–61.9; –1.1)
June to October	–23.1 (–35.0; –11.3)	–21.9 (–46.8; +3.0)	/	–20.6 (–100.0; +184.0)
Geographic areas				
North America	–40.4 (–57.2; –23.6)	/	/	–30.7 (–44.3; –17.0)
Europe	–39.0 (–46.7; –31.3)	/	/	–16.6 (–23.1; –10.1)
Latin America	–34.4 (–53.1; –15.6)	/	/	/
Asia	–37.8 (–50.6; –25.1)	/	/	–40.0 (–52.0; –28.0)
Study setting				
Clinic-based	–39.5 (–46.6; –32.3)	/	/	–25.4 (–34.1; –16.7)
Population-based	–36.9 (–44.1; –29.6)	/	/	–26.9 (–34.0; –19.7)

**Table 2:** Adjusted differences based on multivariate linear regression analysis for overall cancer patients’ visits by type, period, geographic area, and study setting

	Coefficient (95% CI)
Characteristic	Overall visits
Type of visit	
First visits	[Reference]
Follow-up visits	–15.2% (–31.2; 0.70)
Period (2020)	
January and February	[Reference]
March	+7.2% (–14.1; 28.4)
April	–8.2% (–32.7; 16.4)
May	–17.6% (–45.2; 10.0)
June to October	+4.5% (–23.1; 32.1)
Geographic area	
North America	[Reference]
Europe	+3.4% (–22.6; 29.4)
Latin America	+0.4% (–23.8; 24.8)
Asia	–3.0% (–30.7; 24.8)
Study setting	
Clinic-based	[Reference]
Population-based	+12.1% (–6.4; 30.6)

Supplementary Fig. 3 reports the temporal distribution by publication date of the studies included in the analysis of the present outcome.

### Admissions

The mean weighted relative change of oncologic admissions through January–October 2020 was –26.3% (95% CI –31.4; –21.1), with a nadir in May (–31.5%, 95% CI –61.9; –1.1) (Table 1, Fig. 2).

In the stratified analysis by geographic area, whose distribution is shown in Supplementary Fig. 2, Asia showed the greatest decrease (–40.0%, 95% CI –52.0; –28.0) and Europe the smallest one (–16.6%, 95% CI –23.1; –10.1); this difference was confirmed in the multivariate linear regression model analysis (Table 3).

**Table 3:** Adjusted differences based on multivariate linear regression analysis for overall cancer patients’ hospital admissions by period, geographic area, and study setting

	Coefficient (95% CI)
Characteristic	Admissions
Period (2020)	
January and February	[Reference]
March	–6.4% (–17.9; 5.2)
April	–8.5% (–22.0; 5.1)
May	–11.5% (–27.9; 4.8)
June to October	+10.0% (–10.7; 30.8)
Geographic area	
North America	[Reference]
Europe	+18.1% (7.1; 29.1)
Asia	–5.2% (–20.0; 9.7)
Study setting	
Clinic-based	[Reference]
Population-based	+4.8% (–2.7; 12.2)

Results for clinic-based and population-based studies were similar: –25.4% (95% CI –34.1; –21.1) and –26.9% (95% CI –34.0; –19.7), respectively.

The distribution by publication date is shown in Supplementary Fig. 4.

No evidence of publication bias either qualitatively according to funnel plot asymmetry or quantitatively with the Egger regression test (Egger et al. 1997) was identified (*P* values = .69 for oncologic visits and .67 for oncologic admissions); no significant heterogeneity was found among studies using I^2^ test (I^2^ < .001).

## Discussion

Our results showed that both visits and hospital admissions for cancer patients had a significant decrease during the first 10 months of 2020. Overall visits for cancer patients decreased consistently more than hospital admission, maintaining lower values in each period analyzed.

The results on the temporal trend of oncologic visits along January–October 2020 showed a nadir in April 2020, consistent with what emerged from a similar analysis of cancer care during the COVID-19 pandemic (Teglia et al. 2022a, b, Angelini et al. 2023). Although the difference between follow-up visits and first visits was not statistically significant in the multivariate linear regression analysis, a greater decrease was found for the first one compared to the other, indicating that patients or healthcare professionals preferred to postpone programmed follow-up visits rather than first visits, which are usually more compelling.

During the acute phase of the COVID-19 pandemic, anxiety and fear of infection were accounted for as the third most frequent reason for postponement or cancellation of chemotherapy appointments, after neutropenia and thrombocytopenia (Karacin et al. 2020). An Italian survey (Gebbia et al. 2020) revealed an association between frequency of queries to delay medical appointments with older age (>75 years old) and poor performance status of patients, showing that more fragile patients and their families were the most concerned.

The introduction of telemedicine (Karacin et al. 2020) demonstrated to be associated with a significant decrease in postponement of appointments, indicating that sharing information with the public can help to alleviate concerns. Virtual visits or video consultations have been used as a substitute for face-to-face visits during the pandemic (Van de Poll-Franse et al. 2021), in order to eliminate the risk of contracting COVID-19 during hospital visits, while also reducing crowding within medical centers.

Straight-to-test is another channel that general practitioners (GP) can use for patients with suspected oncologic disease (O’Donohoe et al. 2021). For this reason, educational media campaigns to urge people to seek help when needed are vital to prevent patients delaying or avoiding contact with a GP (Abdellatif et al. 2021). In fact, the emphasis on social distancing might have inappropriately convinced patients to avoid in-person medical care, in addition to the suspension of medical services, which made it more complex for outpatients to get an appointment (Reichardt et al. 2021).

The reduction of oncologic visits found in our analysis is likely to be associated with a longer time interval from symptom onset to referral and to diagnosis, a phenomenon reported elsewhere (Abdellatif et al. 2021), that combined with the decrease in cancer screening programs (Teglia et al. 2022a), in visits by general practitioners and in diagnostic procedures (Angelini et al. 2023), is expected to lead, according to the UK Health and Social Care Commettee (2022), to underdiagnosis and undertreatment of cancer and additional cancer deaths (Fonseca et al. 2021). Hospital admissions of oncologic patients showed a global significant decrease, reaching a nadir in May 2020. The difficulty in reaching hospitals for appropriate care (Akhtar et al. 2021), the inability of hospitals to deliver services because of the reallocation of resources to contrast the COVID-19 pandemic, and the consequent reorganization of hospital departments likely impacted on this oncologic service (Patt et al. 2020).

In the analysis by geographic area, the results on hospital admissions for cancer patients showed more heterogeneity in the decline than those on oncologic visits, with the greatest decrease in Asia (–40.0%) and the smallest in Europe (–16.6%). The multivariate linear regression analysis confirmed these results, which are coherent to the ones of a previous meta-analysis on cancer treatment during the COVID-19 pandemic (Teglia et al. 2022b), in which studies from Asia showed the greatest decrease for medical, surgical, and overall cancer treatments, while those from Europe had a smaller decrease for the same outcomes and a faster recovery to pre-pandemic levels. The similarity of the results of the two analyses could be explained by the decrease in cancer screening tests and diagnosis, shown in other previous analyses by our group (Teglia et al. 2022a, Angelini et al. 2023), which probably reduced to a similar extent the number of oncologic patients to be both treated and admitted to medical facilities.

Additional factors may also have played a role, for example, the decrease in the volume of patients with cancer admitted to the emergency department or transferred from outside hospitals (Zubiri et al. 2021) and the decrease in available places due to the use of single-bed rooms in order to reduce nosocomial infections (Gregersen et al. 2021).

Some studies (Tolone et al. 2020, Gambardella et al. 2020) report the use of pre-admission telephone triage to identify COVID-19 positive patients and to assess their risk, which could be crucial considering the frailty of cancer patients.

Our review has some limitations. First, considerable heterogeneity between countries and type of structures was present in terms of service accessibility and participation of the target population, lockdown measures, and incidence and temporal trends of COVID-19 infection; all of these factors could not be considered in our statistical analysis. While other possible risks of bias (including the ones mentioned above) were evaluated by the studies included in our meta-analysis, because they measured the variation of the actual number of patients accessing medical services, only the publication bias was assessed in this article since it did not concern information on patients. Second, the attribution of an observation to one period was based on its beginning date, which might have led to nondifferential misclassification, thus reducing differences between periods. Unlike the pandemic periods, which were divided into 5-time intervals, it was not possible to define groups of pre-pandemic periods to use as reference due to the great heterogeneity among the studies included in the review. Last, in some studies (*N* = 11), we had to impute the number of daily events in order to calculate the weight.

The generalizability of our results is increased by the large number of studies included (*N* = 46), conducted in 24 countries over the world and by the inclusion of the great majority of the articles that are supposed to be published on this topic, according to the distribution of the studies by publication date, represented in Supplementary Figs. 3 and 4.

The COVID-19 pandemic was an unprecedented health crisis that had an impact on all healthcare services in 2020 and will probably continue to affect them in the years ahead. The present systematic review and meta-analysis found a significant decrease in the number of oncologic visits and hospital admissions between January and October 2020 compared to the pre-pandemic period, with potential negative effects on patients’ health and survival. Future studies are needed to fully clarify the long-term implications of this phenomenon and to adopt adequate public health strategies.

## Supplementary information

ESM 1 (DOCX 668 kb)

## Data Availability

Tables including the main information about all the studies considered in the article (title, authors, year of publication, country, compared periods, setting, quality assessment score) are available in the supplementary material. The study protocol and the search terms for our research are available in the supplementary material. No unpublished material or study has been used in our article.
