# Supplementary material for: Decrease of visits and hospital admissions for cancer patients during the COVID-19 pandemic. A systematic review and meta-analysis
Source: Z Gesundh Wiss. 2023 Mar 16:1–7. Online ahead of print. doi: 10.1007/s10389-023-01857-w (PMC10018603; doi:10.1007/s10389-023-01857-w)
Supplement: Supplementary file 1 — (DOCX 668 kb) [file 10389_2023_1857_MOESM1_ESM.docx]

**Supplementary Materials**

**Supplementary Table 1 Characteristics of studies selected for cancer patient’s medical visits**

| **Ref** | **Country** | **Contrast period** | **Period on exam** | **Site of cancer** | **Setting and database** | **Quality assessment score** |
| --- | --- | --- | --- | --- | --- | --- |
| Ak et al. 2020) | Turkey | March 10, 2019 – May 10, 2019 | March 10, 2020 – May 10, 2020 | Miscellaneous | Hospital Registration System of Istanbul University, Institute of Oncology | 10 |
| Karacin et al. 2021 | Turkey | March 15, 2019 – June 1, 2019 | March 15, 2020 – June 1, 2020 | Miscellaneous | Ankara City Hospital, Ankara Diskapi Yildirim Beyazit Training and Research Hospital, and Gulhane Training and Research Hospital | 10 |
|  | Turkey | March 15, 2019 – June 1, 2019 | March 15, 2020 – June 1, 2020 | Miscellaneous | Ankara City Hospital, Ankara Diskapi Yildirim Beyazit Training and Research Hospital, and Gulhane Training and Research Hospital |  |
| Gazivoda et al. 2021 | USA | January 1, 2020 – February 29, 2020 | March 1, 2020 – April 30, 2020 | Miscellaneous | Rutgers Cancer Institute of New Jersey | 10 |
|  | USA | January 1, 2020 – February 29, 2020 | March 1, 2020 – April 30, 2020 | Gynecologic | Rutgers Cancer Institute of New Jersey | 10 |
|  | USA | January 1, 2020 – February 29, 2020 | March 1, 2020 – April 30, 2020 | Gastrointestinal | Rutgers Cancer Institute of New Jersey | 10 |
|  | USA | January 1, 2020 – February 29, 2020 | March 1, 2020 – April 30, 2020 | Skin cancer | Rutgers Cancer Institute of New Jersey | 10 |
|  | USA | January 1, 2020 – February 29, 2020 | March 1, 2020 – April 30, 2020 | Breast | Rutgers Cancer Institute of New Jersey | 10 |
|  | USA | January 1, 2020 – February 29, 2020 | March 1, 2020 – April 30, 2020 | Endocrine | Rutgers Cancer Institute of New Jersey | 10 |
| Zadnik et al 2020 | Slovenia | November 1, 2019 – February 29, 2020 | April 1, 2020 – April 30, 2020 | Miscellaneous* | Institute of Oncology Ljubljana | 8 |
|  | Slovenia | November 1, 2019 – February 29, 2020 | April 1, 2020 – April 30, 2020 | Miscellaneous* | Institute of Oncology Ljubljana | 8 |
|  | Slovenia | November 1, 2019 – February 29, 2020 | April 1, 2020 – April 30, 2020 | Miscellaneous* | Institute of Oncology Ljubljana | 8 |
|  | Slovenia | November 1, 2019 – February 29, 2020 | April 1, 2020 – April 30, 2020 | Miscellaneous* | Institute of Oncology Ljubljana | 8 |
|  | Slovenia | November 1, 2019 – February 29, 2020 | April 1, 2020 – April 30, 2020 | Miscellaneous* | Institute of Oncology Ljubljana | 8 |
|  | Slovenia | November 1, 2019 – February 29, 2020 | April 1, 2020 – April 30, 2020 | Miscellaneous* | Institute of Oncology Ljubljana | 8 |
|  | Slovenia | November 1, 2019 – February 29, 2020 | April 1, 2020 – April 30, 2020 | Miscellaneous* | Institute of Oncology Ljubljana | 8 |
| Ahmad et al. 2020 | Saudi Arabia | December 1, 2019 – February 29, 2020 | March 01, 2020 - May 31, 2020 | Miscellaneous | Tertiary care pediatric oncology and stem cell transplant unit in Riyadh | 8 |
| Ranganatham et al. 2021 | India | March 1, 2019 – May 31, 2019 | March 1, 2020 – May 31, 2020 | Miscellaneous | National Cancer Grid of India | 10 |
|  | India | March 1, 2019 – May 31, 2019 | March 1, 2020 – May 31, 2020 | Miscellaneous | National Cancer Grid of India | 10 |
| Patt et al. 2020 | USA | March 1, 2019 – March 31, 2019 | March 1, 2020 – March 31, 2020 | Miscellaneous* | Proprietary provider clearinghouse registry | 9.5 |
|  | USA | April 1, 2019 – April 30, 2019 | April 1, 2020 – April 30, 2020 | Miscellaneous* | Proprietary provider clearinghouse registry | 9.5 |
|  | USA | May 1, 2019 – May 31, 2019 | May 1, 2020 – May 31, 2020 | Miscellaneous* | Proprietary provider clearinghouse registry | 9.5 |
|  | USA | June 1, 2019 – July 31, 2019 | June1, 2020 – July 31, 2020 | Miscellaneous* | Proprietary provider clearinghouse registry | 9.5 |
| Abdellatif et al. 2021 | Brazil | March 1, 2019 – June 30, 2019 | March 1, 2020 – June 30, 2020 | Colorectal | Kettering General Hospital | 7.5 |
| Aguiar et al 2021 | Brazil | March 1, 2019 – July 31, 2019 | March 1, 2020 – July, 31, 2020 | Colorectal | AC Camargo Cancer Centre, Sao Paulo, Brazil | 7.5 |
| Araujo et al. 2020 | Brazil | March 1, 2019 – May 31, 2019 | March 1, 2020 – May31, 2020 | Miscellaneous | Electronic Health database | 7.5 |
| Wang et al. 2021 | UK | March 1, 2019 – June, 30 2019 | March 1, 2020 – June, 30 2020 | Miscellaneous | Ocular Oncology centres in the UK | 8 |
|  | UK | March 1, 2019 – June, 30 2019 | July 1, 2020 – October 31, 2020 | Miscellaneous | Ocular Oncology centres in the UK | 8 |
| Khan et al. 2021 | USA | March 15, 2019 – July 15, 2019 | March 15, 2020 – July 15, 2020 | Gastrointestinal | TriNetX (Cambridge, MA) database | 8.5 |
| Suárez et al. 2021 | Spain | March 14, 2019 – June 20, 2019 | March 14, 2020 – June 20, 2020 | Colorectal | Complejo Hospitalario de Navarra (CHN) | 8.5 |
|  | Spain | March 14, 2019 – June 20, 2019 | March 14, 2020 – June 20, 2020 | Colorectal | Complejo Hospitalario de Navarra (CHN) | 8.5 |
| Nabhen et al. 2020 | Brazil | March 20, 2019 – June 30, 2019 | March 20, 2020 – June 30, 2020 | Miscellaneous | CACON cancer tracking database | 7 |
| Castillo et al. 2021 | Uruguay | March 13, 2019 - June 30, 2019 | March 13, 2020 – June 30, 2020 | Breast | Oncology Electronic Health Record (Historia Clínica Electrónica Oncológica [HCEO]) | 7.5 |
| Koca et al. 2021 | Turkey | March 11, 2019 – March 10, 2020 | March 11, 2020 – March 11, 2021 | Breast | The Tokat Gaziosmanpasa University (TOGU) Faculty of Medicine General Surgery Clinic | 9 |
| Morris et al. 2021 | UK | January 1, 2019 – December 31, 2019 | January 1, 2020 –February 29, 2020 | Colorectal* | National Health Service (NHS) population-based datasets | 7 |
|  | UK | January 1, 2019 – December 31, 2019 | March 1, 2020 – March 31, 2020 | Colorectal | National Health Service (NHS) population-based datasets | 7 |
|  | UK | January 1, 2019 – December 31, 2019 | April 1, 2020 – April 30, 2020 | Colorectal | National Health Service (NHS) population-based datasets | 7 |
|  | UK | January 1, 2019 – December 31, 2019 | May 1, 2020 – May 31, 2020 | Colorectal | National Health Service (NHS) population-based datasets | 7 |
|  | UK | January 1, 2019 – December 31, 2019 | June 1, 2020 –October 31, 2020 | Colorectal* | National Health Service (NHS) population-based datasets | 7 |
| Khazaeipour et al. 2021 | Iran | February 20, 2019 – May 20, 2019 | February 20, 2020 – May 20, 2020 | Gynecologic | The information technology (IT) department hospital of Tehran University of Medical Sciences (TUMS) | 9 |
| Basu et al. 2021 | Bangladesh | January 1, 2019 –April 30, 2019 | January 1, 2020 –April 30, 2020 | Cervix | Cancer screening registry | 7.5 |
|  | Bangladesh | May 1, 2019 – August 31, 2019 | May 1, 2020 – August 31, 2020 | Cervix | Cancer screening registry | 7.5 |
|  | Bangladesh | Septmeber 1, 2019 – December 31, 2019 | Septmeber 1, 2020 – December 31, 2020 | Cervix | Cancer screening registry | 7.5 |
| Rich et al. 2021 | UK | January 1, 2019 – May 31, 2019 | January 1, 2020 – May 31, 2020 | Skin cancer | Cancer Research UK | 7 |
| Penel et al. 2021 | France | January 20, 2020 – March 15, 2020 | March 16, 2020 – May 10, 2020 | Miscellaneous | The Oscar Lambret Cancer center (Comprehensive Cancer Center of Northern France) | 9 |
|  | France | January 20, 2020 – March 15, 2020 | May 11, 2020 – July 06, 2020 | Miscellaneous | The Oscar Lambret Cancer center (Comprehensive Cancer Center of Northern France) | 9 |
| Longo et al. 2021 | Italy | October 01, 2019 – February 29, 2020 | March 01, 2020 – July 31, 2020 | Miscellaneous | “Casa Sollievo della Sofferenza” hospital | 7.5 |
| Palluzzi et al. 2021 | Italy | February 01, 2019 – April 30, 2019 | February 01, 2020 – April 30, 2020 | Gynecologic | Fondazione Policlinico Agostino Gemelli-IRCCS, Rome | 9.5 |
| Rasschaert et al. 2021 | Belgium | March 17, 2019 – August 28, 2019 | March 17, 2020 – August 28, 2020 | Miscellaneous | The Antwerp University Hospital Cancer Center (MOCA) | 7.5 |
| Mallick et al. 2021 | India | January 1, 2019 – May 16, 2019 | January 1, 2020 – May 16, 2020 | Miscellaneous | The electronic hospital information system (HIS) | 8.5 |
|  | India | January 1, 2019 – May 16, 2019 | January 1, 2020 – May 16, 2020 | Breast* | The electronic hospital information system (HIS) | 8.5 |
|  | India | January 1, 2019 – May 16, 2019 | January 1, 2020 – May 16, 2020 | Lung* | The electronic hospital information system (HIS) | 8.5 |
|  | India | January 1, 2019 – May 16, 2019 | January 1, 2020 – May 16, 2020 | Prostate* | The electronic hospital information system (HIS) | 8.5 |
| Minichsdorfer et al. 2021 | Austria | January 1, 2019 – May 31, 2019 | January 1, 2020 – May 31, 2020 | Miscellaneous | Electronic medical record (EMR) | 8 |
| Buttiron et al. 2021 | Italy | January 1, 2019 – December 31, 2019 | January 1, 2020 – December 31, 2020 | Miscellaneous | Healthcare Professions Structure, Galliera Hospital, Genova, Italy | 8 |
| Kuzuu et al. 2021 | Japan | July 1, 2017 – February 29, 2020 | March 1, 2020 – December 31, 2020 | Gastrointestinal | Yokohama City University Hospital and the National Hospital Organization Yokohama Medical Center | 8 |
|  | Japan | July 1, 2017 – February 29, 2020 | March 1, 2020 – December 31, 2020 | Gastrointestinal | Yokohama City University Hospital and the National Hospital Organization Yokohama Medical Center | 8 |
| Pikkel et al. 2021 | Israel | March 14, 2019 – April 30, 2019 | March 14, 2020 – April 30, 2020 | Melanoma | Rambam Health Care Campus (RHCC) | 8 |
|  | Israel | March 14, 2019 – April 30, 2019 | March 14, 2020 – April 30, 2020 | Melanoma | Rambam Health Care Campus (RHCC) | 8 |
| Gathani et al. 2021 | England | January 01, 2019 – J February 28, 2019 | January 01, 2020 – February 29, 2020 | Breast* | National Health Service (NHS) cancer service activity | 8.5 |
|  | England | March 01, 2019 – March 31, 2019 | March 01, 2020 – March 31, 2020 | Breast | National Health Service (NHS) cancer service activity | 8.5 |
|  | England | April 01, 2019 – April 30, 2019 | April 01, 2020 – April 30, 2020 | Breast | National Health Service (NHS) cancer service activity | 8.5 |
|  | England | May 01, 2019 – May 31, 2019 | May 01, 2020 – May 31, 2020 | Breast | National Health Service (NHS) cancer service activity | 8.5 |
|  | England | June 01, 2019 – June 30, 2019 | June 01, 2020 – June 30, 2020 | Breast | National Health Service (NHS) cancer service activity | 8.5 |
| Vazquez et al. 2021 | Uruguay | March 16, 2019 – April 30, 2019 | March 16, 2020 – April 30, 2020 | Miscellaneous* | Public Provider | 9.5 |
|  | Uruguay | May 01, 2019 – May 31, 2019 | May 01, 2020 – May 31, 2020 | Miscellaneous | Public Provider | 9.5 |
|  | Uruguay | June 01, 2019 – June 30, 2019 | June 01, 2020 – June 30, 2020 | Miscellaneous | Public Provider | 9.5 |
|  | Chile | April 01, 2019 – April 30, 2019 | April 01, 2020 – April 30, 2020 | Miscellaneous | National cancer institute | 9.5 |
|  | Chile | May 01, 2019 – May 31, 2019 | May 01, 2020 – May 31, 2020 | Miscellaneous | National cancer institute | 9.5 |
|  | Chile | June 01, 2019 – June 30, 2019 | June 01, 20120 – June 30, 2020 | Miscellaneous* | National cancer institute | 9.5 |
|  | Mexico | April 01, 2019 – April 30, 2019 | April 01, 2020 – April 30, 2020 | Miscellaneous* | General Hospital and National Cancer institute | 9.5 |
|  | Mexico | May 01, 2019 – May 31, 2019 | May 01, 2020 – May 31, 2020 | Miscellaneous* | General Hospital and National Cancer institute | 9.5 |
|  | Mexico | June 01, 2019 – June 30, 2019 | June 01, 20120 – June 30, 2020 | Miscellaneous* | General Hospital and National Cancer institute | 9.5 |
|  | Mexico | April 01, 2019 – April 30, 2019 | April 01, 2020 – April 30, 2020 | Miscellaneous* | General Hospital and National Cancer institute | 9.5 |
|  | Mexico | May 01, 2019 – May 31, 2019 | May 01, 2020 – May 31, 2020 | Miscellaneous* | General Hospital and National Cancer institute | 9.5 |
|  | Mexico | June 01, 2019 – June 30, 2019 | June 01, 20120 – June 30, 2020 | Miscellaneous* | General Hospital and National Cancer institute | 9.5 |
|  | Colombia | March 16, 2019 – April 30, 2019 | March 16, 2020 – April 30, 2020 | Miscellaneous* | Private Provider | 9.5 |
|  | Colombia | May 01, 2019 – May 31, 2019 | May 01, 2020 – May 31, 2020 | Miscellaneous* | Private Provider | 9.5 |
|  | Colombia | June 01, 2019 – June 30, 2019 | June 01, 2020 – June 30, 2020 | Miscellaneous* | Private Provider | 9.5 |
| Maganty et al. 2021 | USA | December 17, 2019 – March 16, 2020 | March 17, 2020 – June 17, 2020 | Genito- urinary | Oncology department in Pittsburg | 9 |
|  | USA | December 17, 2019 – March 16, 2020 | March 17, 2020 – June 17, 2020 | Prostate | Oncology department in Pittsburg | 9 |
|  | USA | December 17, 2019 – March 16, 2020 | March 17, 2020 – June 17, 2020 | Bladder | Oncology department in Pittsburg | 9 |
|  | USA | December 17, 2019 – March 16, 2020 | March 17, 2020 – June 17, 2020 | Kidney | Oncology department in Pittsburg | 9 |
| Montesi et al. 2020 | Italy | February 1, 2019 – March 31, 2019 | February 1, 2020 – March 31, 2020 | Miscellaneous | Radiotherapy department | 7 |
| Salem et al. 2020 | Lebanon | March 11, 2019 – May 18, 2019 | March 09, 2020 – May 17, 2020 | Breast | The University hospital institution (Hôtel-Dieu de France) | 7.5 |
| Mahase et al. 2020 | UK | April 1, 2019 – April 30, 2019 | April 1, 2020 – April 30, 2020 | Miscellaneous | NHS England | 7 |
| Earnshaw et al. 2020 | UK | February 1, 2019 – April 30, 2019 | February 1, 2020 – April 30, 2020 | Skin cancer* | The histopathology labs and the Cancer Tracking Service at Salford Royal Hospital | 7 |
| Brugel et al. 2021 | France | March 1, 2019 – May 31, 2019 | March 1, 2020 – May 31, 2020 | Miscellaneous* | University Hospital and Godinot Cancer Institute in Reims, Cancer Coordination Center (3C), Pasteur Hospital, Colmar | 9.5 |
|  | France | March 1, 2019 – May 31, 2019 | March 1, 2020 – May 31, 2020 | Miscellaneous* | University Hospital and Godinot Cancer Institute in Reims, Cancer Coordination Center (3C), Pasteur Hospital, Colmar | 9.5 |

*calculated value using data present in the article

**Supplementary Table 2 Characteristics of studies selected for cancer patient’s hospital admission**

| **Ref** | **Country** | **Contrast period** | **Period on exam** | **Site of cancer** | **Setting and database** | **Quality assessment score** |
| --- | --- | --- | --- | --- | --- | --- |
| Ak et al. 2020) | Turkey | March 10, 2019 – May 10, 2019 | March 10, 2020 – May 10, 2020 | Miscellaneous | Hospital Registration System of Istanbul University, Institute of Oncology | 10 |
| Ahmad et al. 2020 | Saudi Arabia | December 1, 2019 – February 29, 2020 | March 1, 2020 - May 31, 2020 | Miscellaneous | Tertiary care pediatric oncology and stem cell transplant unit in Riyadh | 8 |
| Ranganatham et al. 2021 | India | March 1, 2019 – May 31, 2019 | March 1, 2020 – May 31, 2020 | Miscellaneous | National Cancer Grid of India | 10 |
| Patt et al. 2020 | USA | March 1, 2019 – March 31, 2019 | March 1, 2020 – March 31, 2020 | Miscellaneous* | Proprietary provider clearinghouse registry | 9.5 |
|  | USA | April 1, 2019 – April 30, 2019 | April 1, 2020 – April 30, 2020 | Miscellaneous* | Proprietary provider clearinghouse registry | 9.5 |
|  | USA | May 1, 2019 – May 31, 2019 | May 1, 2020 – May 31, 2020 | Miscellaneous* | Proprietary provider clearinghouse registry | 9.5 |
|  | USA | June 1, 2019 – July 31, 2019 | June1, 2020 – July 31, 2020 | Miscellaneous* | Proprietary provider clearinghouse registry | 9.5 |
| Mafra da Costa et al. 2021 | Brazil | March 1, 2019 – July 31, 2019 | March 1, 2020 – July 31, 2020 | Misccellaneous | Brazil’s National Public Health System (Sistema Unico de Sade - SUS) | 7.5 |
|  | Brazil | March 1, 2019 – July 31, 2019 | March 1, 2020 – July 31, 2020 | Misccellaneous | Brazil’s National Public Health System (Sistema Unico de Sade - SUS) | 7.5 |
| Araujo et al. 2020 | Brazil | March 1, 2019 – May 31, 2019 | March 1, 2020 – May31, 2020 | Miscellaneous | Electronic Health database | 7.5 |
| Tlemsani et al. 2021 | France | March 17, 2019 – April 30, 2019 | March 17, 2020 – April 30, 2020 | Miscellaneous* | Cochin Hospital, University Hospital | 8 |
| Khazaeipour et al. 2021 | Iran | February 20, 2019 – May 20, 2019 | February 20, 2020 – May 20, 2020 | Gynecologic | The information technology (IT) department hospital of Tehran University of Medical Sciences (TUMS) | 9 |
| Penel et al. 2021 | France | January 20, 2020 – March 15, 2020 | March 16, 2020 – May 10, 2020 | Miscellaneous | The Oscar Lambret Cancer center (Comprehensive Cancer Center of Northern France) | 9 |
|  | France | January 20, 2020 – March 15, 2020 | May 11, 2020 – July 06, 2020 | Miscellaneous | The Oscar Lambret Cancer center (Comprehensive Cancer Center of Northern France) | 9 |
|  | France | January 20, 2020 – March 15, 2020 | March 16, 2020 – May 10, 2020 | Miscellaneous | The Oscar Lambret Cancer center (Comprehensive Cancer Center of Northern France) | 9 |
|  | France | January 20, 2020 – March 15, 2020 | May 11, 2020 – July 06, 2020 | Miscellaneous | The Oscar Lambret Cancer center (Comprehensive Cancer Center of Northern France) | 9 |
| Longo et al. 2021 | Italy | March 1, 2019 – November 30, 2020 | March 1, 2020 – November 30, 2020 | Head and Neck | “Casa Sollievo dalla Sofferenza” hospital | 7.5 |
| Palluzzi et al. 2021 | Italy | February 01, 2019 – April 30, 2019 | February 01, 2020 – April 30, 2020 | Gynecologic | Fondazione Policlinico Agostino Gemelli-IRCCS, Rome | 9.5 |
| Rasschaert et al. 2021 | Belgium | March 17, 2019 – August 28, 2019 | March 17, 2020 – August 28, 2020 | Miscellaneous* | The Antwerp University Hospital Cancer Center (MOCA) | 7.5 |
| Crusz et al. 2021 | UK | April 1, 2019 – April 30, 2019 | April 1, 2020 – April 30, 2020 | Miscellaneous | Royal London Hospital, Newham University Hospital and Whipps Cross University Hospital | 8 |
| Buttiron et al. 2021 | Italy | January 1, 2019 – December 31, 2019 | January 1, 2020 – December 31, 2020 | Miscellaneous | Healthcare Professions Structure, Galliera Hospital, Genova, Italy | 8 |
| Bollmann et al. 2021 | Germany | March 15, 2019 – December 12, 2019 | March 13, 2020 – December 10, 2020 | Miscellaneous* | 86 Helios hospitals in Germany | 8 |
| Li et al. 2020 | China | December 20, 2019 – January 19, 2020 | January 20, 2020 – March 20, 2020 | Stomach | General Surgery Department | 8 |
| Zubiri et al. 2021 | USA | January 1, 2019 – December 31, 2019 | March 1, 2020 – May 31, 2020 | Miscellaneous | Massachusetts General Hospital (MGH) in Boston | 8 |
|  | USA | June 1, 2019 – August 31, 2019 | June 1, 2020 – August 31, 2020 | Miscellaneous | Massachusetts General Hospital (MGH) in Boston | 8 |
| Fonseca et al. 2021 | Brazil | March 1, 2019 – May 31, 2019 | March 1, 2020 – May 31, 2020 | Miscellaneous* | SUS Hospital and Ambulatory Information Systems using the DATASUS (Departamento de Informática do Sistema Único de Saúde) platform | 9 |
| Gonnelli et al. 2020 | Italy | March 9, 2019 – May 31, 2019 | March 9, 2020 – May 31, 2020 | Miscellaneous | University hospital of Pisa | 8 |
| Reichatdt et al. 2021 | Germany | March 13, 2019 – April 28, 2019 | March 13, 2020 – April 28, 2020 | Miscellaneous | 75 Helios Hospitals in Germany | 8 |
|  | Germany | April 29, 2019 – June 14, 2019 | April 29, 2020 – June 14, 2020 | Miscellaneous | 75 Helios Hospitals in Germany | 8 |
|  | Germany | March 13, 2019 – April 28, 2019 | March 13, 2020 – April 28, 2020 | Gastrointestinal | 75 Helios Hospitals in Germany | 8 |
|  | Germany | April 29, 2019 – June 14, 2019 | April 29, 2020 – June 14, 2020 | Gastrointestinal | 75 Helios Hospitals in Germany | 8 |
|  | Germany | March 13, 2019 – April 28, 2019 | March 13, 2020 – April 28, 2020 | Skin cancer | 75 Helios Hospitals in Germany | 8 |
|  | Germany | April 29, 2019 – June 14, 2019 | April 29, 2020 – June 14, 2020 | Skin cancer | 75 Helios Hospitals in Germany | 8 |
|  | Germany | March 13, 2019 – April 28, 2019 | March 13, 2020 – April 28, 2020 | Breast | 75 Helios Hospitals in Germany | 8 |
|  | Germany | April 29, 2019 – June 14, 2019 | April 29, 2020 – June 14, 2020 | Breast | 75 Helios Hospitals in Germany | 8 |
|  | Germany | March 13, 2019 – April 28, 2019 | March 13, 2020 – April 28, 2020 | Gynecologic | 75 Helios Hospitals in Germany | 8 |
|  | Germany | April 29, 2019 – June 14, 2019 | April 29, 2020 – June 14, 2020 | Gynecologic | 75 Helios Hospitals in Germany | 8 |
|  | Germany | March 13, 2019 – April 28, 2019 | March 13, 2020 – April 28, 2020 | Genito-urinary | 75 Helios Hospitals in Germany | 8 |
|  | Germany | April 29, 2019 – June 14, 2019 | April 29, 2020 – June 14, 2020 | Genito- urinary | 75 Helios Hospitals in Germany | 8 |
|  | Germany | March 13, 2019 – April 28, 2019 | March 13, 2020 – April 28, 2020 | Hematologic cancer | 75 Helios Hospitals in Germany | 8 |
|  | Germany | April 29, 2019 – June 14, 2019 | April 29, 2020 – June 14, 2020 | Hematologic cancer | 75 Helios Hospitals in Germany | 8 |

*calculated value using data present in the article

**References**

Abdellatif M, Salama Y, Alhammali T, Eltweri AM. (2021) Impact of COVID-19 on colorectal cancer early diagnosis pathway: retrospective cohort study. *Br J Surg*. 108(4):e146-e147. <https://doi.org/10.1093/bjs/znaa122>

Aguiar S, Riechelmann RP, de Mello CAL, et al. (2021) Impact of COVID-19 on colorectal cancer presentation. *Br J Surg*. 108(2):e81-e82. <https://doi.org/10.1093/bjs/znaa124>

Ahmad N, Essa MF, Sudairy R. (2020) Impact of Covid19 on a tertiary care pediatric oncology and stem cell transplant unit in Riyadh, Saudi Arabia *Pediatr Blood Cancer*. 67(9):e28560. <https://doi.org/10.1002/pbc.28560>

Ak N, Vatansever S. (2020) "Door to Treatment" Outcomes of Cancer Patients during the COVID-19 Pandemic. *Chemotherapy*. 65(5-6):141-146. <https://doi.org/10.1159/000511884>

Araujo SEA, Leal A, Centrone AFY, et al. (2020) Impact of COVID-19 pandemic on care of oncological patients: experience of a cancer center in a Latin American pandemic epicenter. *Einstein (Sao Paulo)*. 19:eAO6282. <https://doi.org/10.31744/einstein_journal/2021AO6282>

Basu P, Lucas E, Zhang L, Muwonge R, Murillo R, Nessa A. (2021) Leveraging vertical COVID-19 investments to improve monitoring of cancer screening programme - A case study from Bangladesh. *Prev Med*. 151:106624. <https://doi.org/10.1016/j.ypmed.2021.106624>

Bollmann A, Hohenstein S, Pellissier V, et al. (2021) Utilization of in- and outpatient hospital care in Germany during the Covid-19 pandemic insights from the German-wide Helios hospital network. *PLoS One*. 16(3):e0249251. Published 2021 Mar 25. <https://doi.org/10.1371/journal.pone.0249251>

Brugel M, Carlier C, Essner C, et al. (2021) Dramatic Changes in Oncology Care Pathways During the COVID-19 Pandemic: The French ONCOCARE-COV Study. *Oncologist*. 26(2):e338-e341. <https://doi.org/10.1002/onco.13578>

Buttiron Webber T, Giuliano S, Patrone C, et al. (2021) Home Se-Cure: A Home Care Service for Cancer Patients during the COVID-19 Pandemic. *Int J Environ Res Public Health*. 18(20):10913. Published 2021 Oct 17. <https://doi.org/10.3390/ijerph182010913>

Castillo C, Camejo N, Amarillo D, et al. (2021) Impact of the COVID-19 pandemic on health care activities at a Uruguayan mastology unit. *J Cancer Res Ther*. 17(2):547-550. <https://doi.org/10.4103/jcrt.JCRT_1689_20>

Crusz SM, Hall PE, Earwicker K, et al. (2021) Providing an acute oncology service during the COVID-19 pandemic. *Clin Med (Lond)*. 21(5):e548-e551. <https://doi.org/10.7861/clinmed.2020-0693>

Earnshaw CH, Hunter HJA, McMullen E, Griffiths CEM, Warren RB. (2020) Reduction in skin cancer diagnosis, and overall cancer referrals, during the COVID-19 pandemic. *Br J Dermatol*. 183(4):792-794. <https://doi.org/10.1111/bjd.19267>

Fonseca GA, Normando PG, Loureiro LVM, et al. (2021) Reduction in the Number of Procedures and Hospitalizations and Increase in Cancer Mortality During the COVID-19 Pandemic in Brazil. *JCO Glob Oncol*. 7:4-9 <https://doi.org/10.1200/GO.20.00471>

Gathani T, Clayton G, MacInnes E, Horgan K. (2021) The COVID-19 pandemic and impact on breast cancer diagnoses: what happened in England in the first half of 2020. *Br J Cancer*. 124(4):710-712. <https://doi.org/10.1038/s41416-020-01182-z>

Gazivoda V, Greenbaum A, Roshal J, et al. (2021) Assessing the immediate impact of COVID-19 on surgical oncology practice: Experience from an NCI-designated Comprehensive Cancer Center in the Northeastern United States. *J Surg Oncol*. 124(1):7-15. <https://doi.org/10.1002/jso.26475>

Gonnelli A, Montrone S, Cocuzza P, et al. (2020) Radiotherapy in the COVID-19 Pandemic Era. *In Vivo*. 34(6):3731-3734 <https://doi.org/10.21873/invivo.12222>

Karacin C, Acar R, Bal O, et al. (2021) "Swords and Shields" against COVID-19 for patients with cancer at "clean" and "pandemic" hospitals: are we ready for the second wave?. *Support Care Cancer*. 29(8):4587-4593. <https://doi.org/10.1007/s00520-021-06001-6>

Khan A, Bilal M, Morrow V, Cooper G, Thakkar S, Singh S. (2021) Impact of the Coronavirus Disease 2019 Pandemic on Gastrointestinal Procedures and Cancers in the United States: A Multicenter Research Network Study. *Gastroenterology*. 160(7):2602-2604.e5. <https://doi.org/10.1053/j.gastro.2021.02.055>

Khazaeipour Z, Razavi E, Pahlevan-Fallahy MT. (2021) Indirect effects of COVID-19 in referring women to gynecologic oncology, perinatology and gynecology clinics in Iran. *Arch Gynecol Obstet*. 304(3):679-686. <https://doi.org/10.1007/s00404-021-06097-5>

Koca B, Yildirim M. (2021) Delay in breast cancer diagnosis and its clinical consequences during the coronavirus disease pandemic. *J Surg Oncol*. 124(3):261-267. <https://doi.org/10.1002/jso.26581>

Kuzuu K, Misawa N, Ashikari K, et al. (2021) Gastrointestinal Cancer Stage at Diagnosis Before and During the COVID-19 Pandemic in Japan. *JAMA Netw Open*. 4(9):e2126334. Published 2021 Sep 1. <https://doi.org/10.1001/jamanetworkopen.2021.26334>

Li YX, He CZ, Liu YC, et al. (2020) The impact of COVID-19 on gastric cancer surgery: a single-center retrospective study. *BMC Surg*. 20(1):222. Published 2020 Oct 2. <https://doi.org/10.1186/s12893-020-00885-7>

Longo F, Trecca EMC, D'Ecclesia A, et al. (2021) Managing head and neck cancer patients during the COVID-19 pandemic: the experience of a tertiary referral center in southern Italy. *Infect Agent Cancer*. 16(1):9. Published 2021 Feb 5. <https://doi.org/10.1186/s13027-021-00352-9>

Mafra da Costa A, Ribeiro AL, Ribeiro AG, et al. (2021) Impact of COVID-19 Pandemic on Cancer-Related Hospitalizations in Brazil. *Cancer Control*. 28:10732748211038736. <https://doi.org/10.1177/10732748211038736>

Maganty A, Yu M, Anyaeche VI, et al. (2021) Referral pattern for urologic malignancies before and during the COVID-19 pandemic. *Urol Oncol*. 39(5):268-276. <https://doi.org/10.1016/j.urolonc.2020.11.027>

Mahase E. (2020) Covid-19: Urgent cancer referrals fall by 60%, showing "brutal" impact of pandemic. *BMJ*. 369:m2386. Published 2020 Jun 12. <https://doi.org/10.1136/bmj.m2386>

Mallick I, Chakraborty S, Baral S, et al. (2021) Prioritizing Delivery of Cancer Treatment During a COVID-19 Lockdown: The Experience of a Clinical Oncology Service in India. *JCO Glob Oncol*. 7:99-107. <https://doi.org/10.1200/GO.20.00433>

Minichsdorfer C, Jeryczynski G, Krall C, et al. (2021) Impact of COVID-19 lockdown on routine oncology versus emergency care at a high volume cancer centre. *Eur J Clin Invest*. 51(8):e13623. <https://doi.org/10.1111/eci.13623>

Montesi G, Di Biase S, Chierchini S, et al. (2020) Radiotherapy during COVID-19 pandemic. How to create a No fly zone: a Northern Italy experience. *Radiol Med*. 125(6):600-603. <https://doi.org/10.1007/s11547-020-01217-8>

Morris EJA, Goldacre R, Spata E, et al. (2021) Impact of the COVID-19 pandemic on the detection and management of colorectal cancer in England: a population-based study. *Lancet Gastroenterol Hepatol*. 6(3):199-208. <https://doi.org/10.1016/S2468-1253(21)00005-4>

Nabhen JJ, Ostroski TKD, Kozonoe MM, Orlandi D, Tormen T, Ioshii SO. (2020) Impact of the COVID-19 pandemic in patient admission to a high-complexity cancer center in Southern Brasil. *Rev Assoc Med Bras (1992)*. 66(10):1361-1365. <https://doi.org/10.1590/1806-9282.66.10.1361>

Palluzzi E, Corrado G, Marchetti C, et al. (2021) Medical treatment of patients with gynecologic cancer during the COVID-19 pandemic. *Int J Gynecol Cancer*. 31(8):1154-1158. <https://doi.org/10.1136/ijgc-2020-002288>

Patt D, Gordan L, Diaz M, et al. (2020) Impact of COVID-19 on Cancer Care: How the Pandemic Is Delaying Cancer Diagnosis and Treatment for American Seniors. *JCO Clin Cancer Inform*. 4:1059-1071. <https://doi.org/10.1136/ijgc-2020-002288>

Penel N, Hammoudi A, Marliot G, et al. (2021) Major impact of COVID-19 national containment on activities in the French northern comprehensive cancer center. *Med Oncol*. 38(3):28. Published 2021 Feb 17. <https://doi.org/10.1007/s12032-021-01467-0>

Pikkel YY, Duek OS, Ben Naftali Y, Link Y, Khayr M, Ullmann Y. (2021) Hidden in plain sight: the (other) danger of COVID-19. *Melanoma Res*. 31(4):389-392. <https://doi.org/10.1097/CMR.0000000000000745>

Ranganathan P, Sengar M, Chinnaswamy G, et al. (2021) Impact of COVID-19 on cancer care in India: a cohort study. *Lancet Oncol*. 22(7):970-976. <https://doi.org/10.1016/S1470-2045(21)00240-0>

Rasschaert M, Vanclooster P, Depauw L, et al. (2021) Meeting the Challenges in Cancer Care Management During the SARS-Cov-2 Pandemic: A Retrospective Analysis. *Cancer Control*. 28:10732748211045275. <https://doi.org/10.1177/10732748211045275>

Reichardt P, Bollmann A, Hohenstein S, et al. (2021) Decreased Incidence of Oncology Admissions in 75 Helios Hospitals in Germany during the COVID-19 Pandemic. *Oncol Res Treat*. 44(3):71-75. <https://doi.org/10.1159/000512935>

Rich H, O'Neill T.(2021) Major fall in urgent skin cancer referrals during the COVID-19 outbreak. *J Plast Reconstr Aesthet Surg*. 74(3):644-710. <https://doi.org/10.1016/j.bjps.2020.10.087>

Salem C, Hajj MA, Kourié H, et al. (2020) Radiology management of a 'breast unit' during COVID-19 pandemic: a single institution experience. *Future Oncol*. 16(35):2917-2922. <https://doi.org/10.2217/fon-2020-0585>

Suárez J, Mata E, Guerra A, et al. (2021) Impact of the COVID-19 pandemic during Spain's state of emergency on the diagnosis of colorectal cancer. *J Surg Oncol*. 123(1):32-36. <https://doi.org/10.1002/jso.26263>

Tlemsani C, Arrondeau J, De Percin S, et al. (2021) Impact of the COVID-19 pandemic on the management of cancer patients: the experience of the cancer outpatients department of a university hospital in Paris. *Clin Med (Lond)*. 21(5):e552-e555. <https://doi.org/10.7861/clinmed.2020-0666>

Vázquez Rosas T, Cazap E, Delgado L, et al. (2021) Social Distancing and Economic Crisis During COVID-19 Pandemic Reduced Cancer Control in Latin America and Will Result in Increased Late-Stage Diagnoses and Expense. *JCO Glob Oncol*. 7:694-703. <https://doi.org/10.1200/GO.21.00016>

Wang H, Elsheikh M, Gilmour K, et al. (2021) Impact of COVID-19 pandemic on eye cancer care in United Kingdom [published correction appears in Br J Cancer. 125(8):1177]. *Br J Cancer*. 124(8):1357-1360. <https://doi.org/10.1038/s41416-021-01274-4>

Zadnik V, Mihor A, Tomsic S, et al. (2020) Impact of COVID-19 on cancer diagnosis and management in Slovenia - preliminary results. *Radiol Oncol*. 54(3):329-334. <https://doi.org/10.2478/raon-2020-0048>

Zubiri L, Rosovsky RP, Mooradian MJ, et al. (2021) Temporal Trends in Inpatient Oncology Census Before and During the COVID-19 Pandemic and Rates of Nosocomial COVID-19 Among Patients with Cancer at a Large Academic Center. *Oncologist*. 26(8):e1427-e1433. <https://doi.org/10.1002/onco.13807>

**Supplementary Figure 1** Representation of geographic areas of the studies included in the analysis for cancer patient’s visits


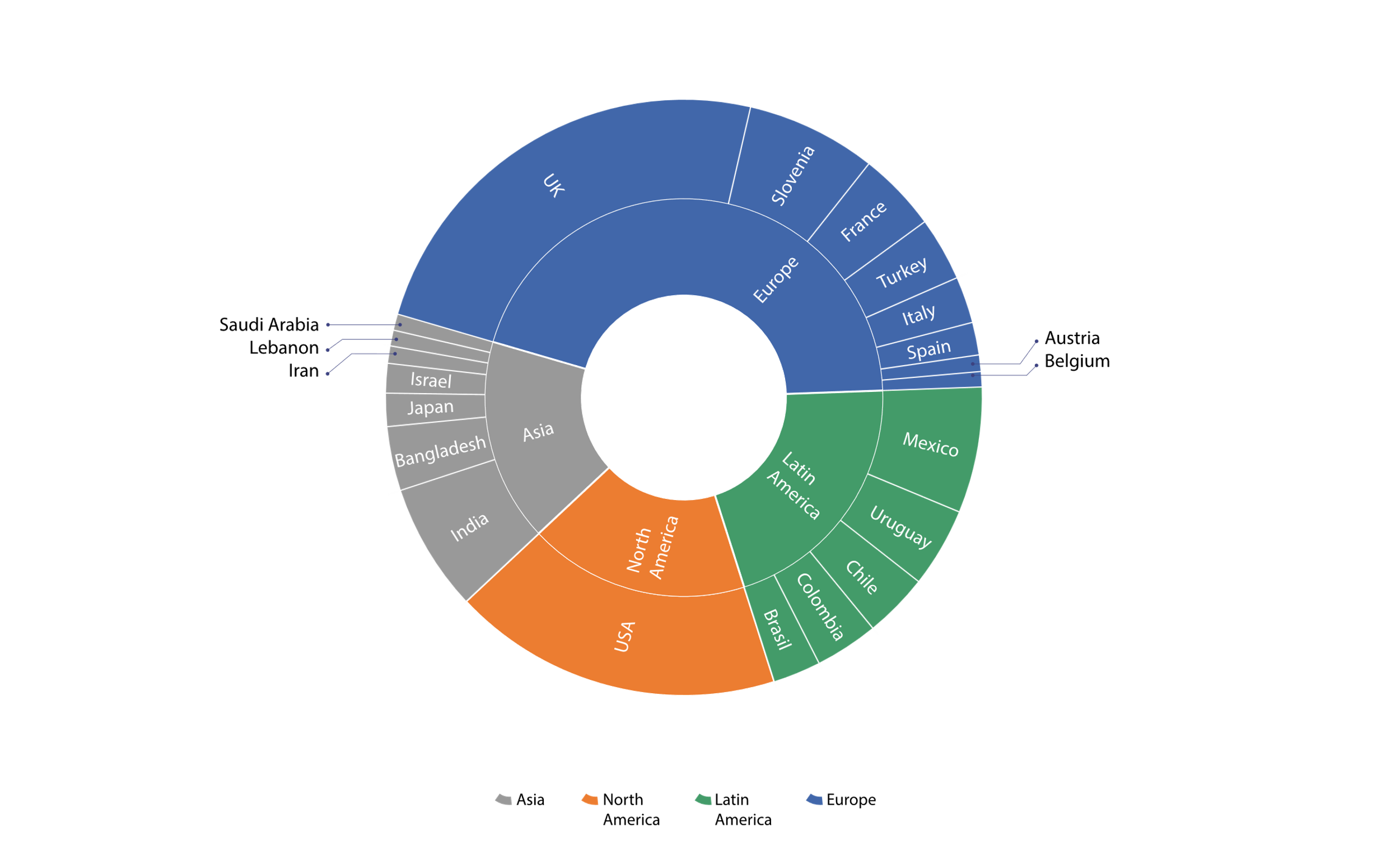


**Supplementary Figure 2** Representation of geographic areas of the studies included in the analysis for cancer patient’s hospital admissions


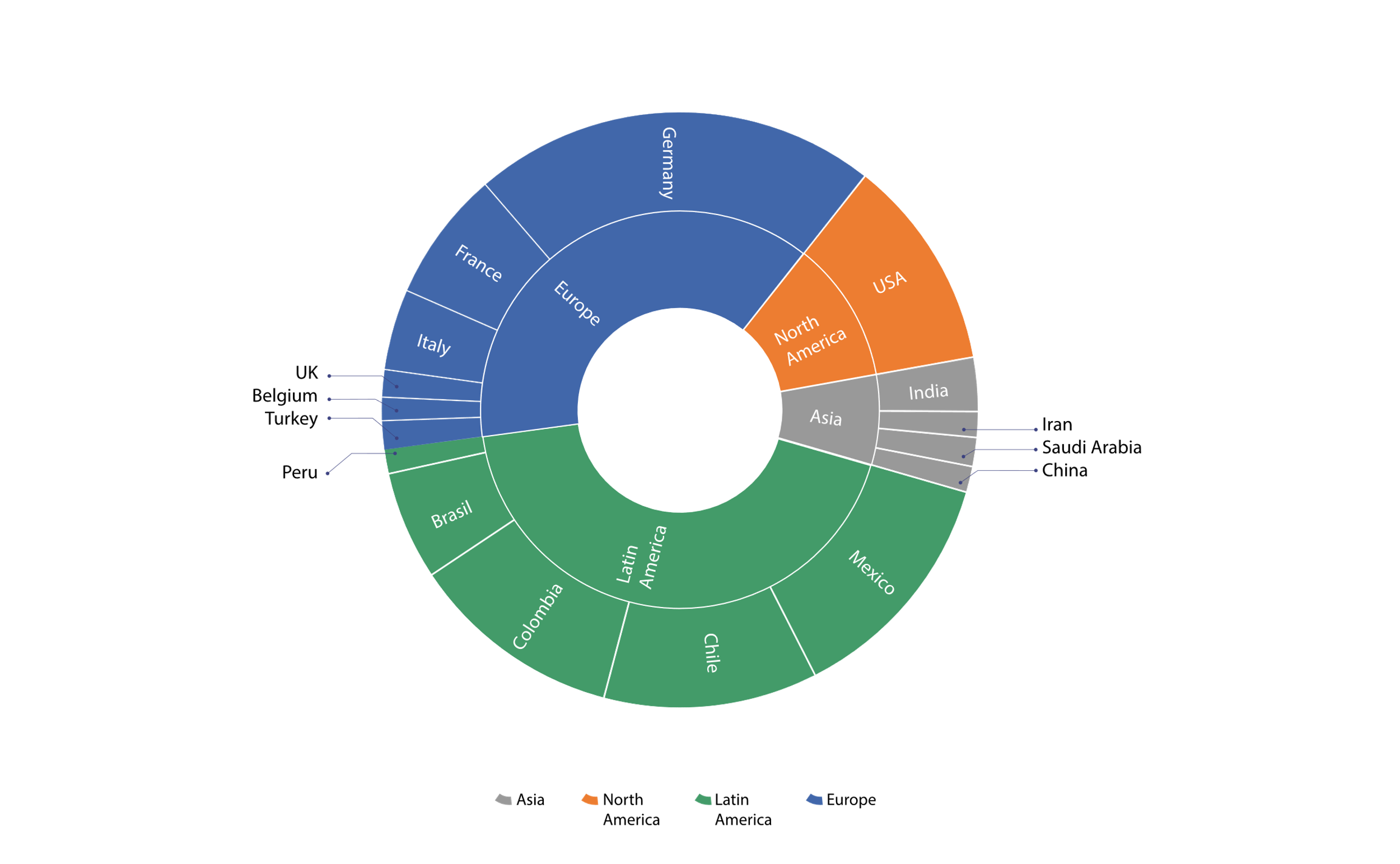


**Supplementary Figure 3**: Representation of the online publication date of the studies included in the analysis for visits for cancer patients by quarter.

**Supplementary Figure 4**: Representation of the online publication date of the studies included in the analysis for hospital admissions of cancer patients by quarter.
